# Groups of familiar male rats form unstable partner preferences when play fighting during the juvenile period

**DOI:** 10.1016/j.isci.2025.112562

**Published:** 2025-05-02

**Authors:** Jackson R. Ham, Sergio M. Pellis

**Affiliations:** 1 Department of Neuroscience, University of Lethbridge, Lethbridge, Alberta T1K 3M4, Canada

**Keywords:** Rodent behavior, Biological sciences, Zoology

## Abstract

Rats are social animals living in large groups. Within these groups, juveniles engage in vigorous rough-and-tumble play. Despite their natural ecology, most current studies of their play behavior involve pairs not groups. To investigate play under more naturalistic settings, we examined the play of eight groups of juvenile male rats, with each group comprising six, same-aged peers that lived together. Each group was tested on multiple days over the peak play period (30–40 postnatal days). On any given day, rats showed partner preferences for certain individuals in the group, however, preferences varied from day to day. Despite changes in partner preferences, rats chose to play with partners that engaged in more turn taking and with partners with whom they had more symmetrical play relationships. That some individuals within the group were consistently preferred as play partners while others were consistently avoided may have developmental consequences, with those who are favored gaining greater benefits from their juvenile play experiences.

## Highlights

• When playing in groups, some rats form partner preferences
• Certain rats are preferred while others are avoided
• Particular individuals are “popular”, and played with consistently
• Preferred rats had more symmetrical play and engaged in more turn taking

## Graphical abstract

**Figure:**
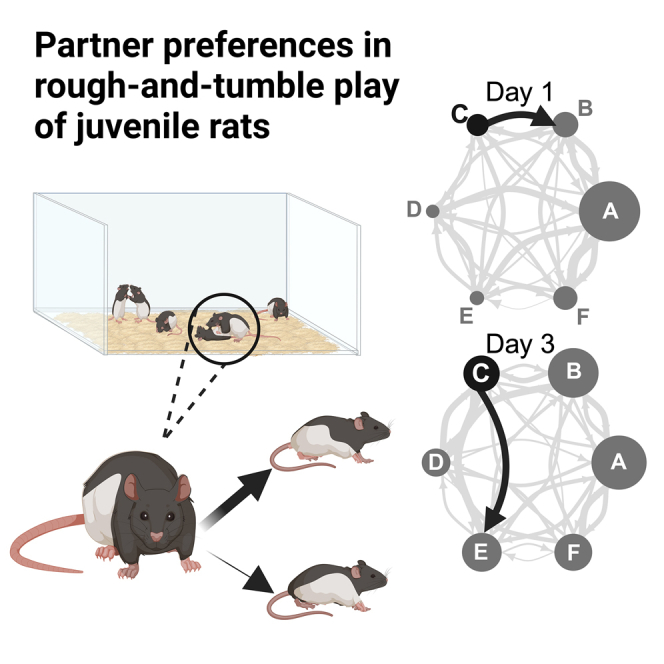

## Introduction

Play fighting or rough-and-tumble play is one of the most commonly reported forms of social play in mammals, especially among juveniles.^1^^,^^2^^,^^3^ Play fighting involves competition to gain a species-specific advantage, which for many species involves contacting a particular body target, but is moderated by some cooperation,^4^^,^^5^ leading to a degree of reciprocity or turn-taking that does not occur in serious fighting.^6^^,^^7^ In laboratory rats (*Rattus norvegicus*), a species in which play fighting has been studied most intensively,^8^^,^^9^^,^^10^^,^^11^ the animals compete to gain access to the nape of their partner’s neck which is nuzzled with the snout if contacted.^12^^,^^13^

Rats are highly motivated to play,^14^^,^^15^ and while most frequent in the juvenile period, such play continues into adulthood.^16^^,^^17^ Because of this propensity to engage in play fighting spontaneously, studies of rats have been instrumental in characterizing the neural circuits and the neurochemical systems involved (e.g.,^8^^,^^11^^,^^18^^,^^19^). In addition, the influence of play fighting on the development of socio-cognitive skills and the anatomy and physiology of the prefrontal cortex has been extensively studied in rats (e.g.,^20^^,^^21^^,^^22^^,^^23^^,^^24^^,^^25^^,^^26^).

While rats are highly motivated to play, individuals show a considerable degree of variation in their play behavior both in the amount of play they initiate and in their style of playing.^10^^,^^27^^,^^28^^,^^29^^,^^30^^,^^31^^,^^32^^,^^33^ For example, based on how much play they initiate, some rats can be classified as “high players” and some as “low players”.^27^^,^^30^ Moreover, pairs of high and low players tend to prefer using different tactics to defend their nape, leading to different styles of play fighting.^10^

For species in which multiple potential play partners are available, not all are equally preferred. In many cases, preferred partners are often age-matched,^34^^,^^35^^,^^36^^,^^37^^,^^38^ same sex,^34^^,^^39^^,^^40^^,^^41^^,^^42^ close relatives,^40^^,^^43^ and of similar dominance rank.^44^^,^^45^ These factors affecting play partner preferences may change with age (e.g., preferring older partners when young, but younger partners when older) and/or the type of social play performed (e.g., sexual play versus rough-and-tumble play).^35^^,^^39^^,^^46^^,^^47^^,^^48^^,^^49^

Rats are highly social, living in large colonies,^50^ with synchronized births from multiple females.^51^ With the average litter size ranging from four to sixteen pups,^50^ juvenile rats have many potential partners with whom to play. Despite this, little is known about potential partner preferences when playing.^28^^,^^52^ As play fighting is typically dyadic,^1^^,^^2^^,^^3^ most research on play in rats has been tested using pairs of rats.^9^ But given the diversity in playfulness among juvenile rats, not all peers may be equally attractive play partners when available in a group context. For example, a high playing rat might prefer to play with a high playing rat, or rats may prefer to play with individuals that complement their own play style. Thus, when we select pairs for testing in dyads, we could be constraining rats to play with incompatible partners. That is, if the rats were given a choice, there may be partners with whom they would not play in a group.

Given the individual variation in play, and that rats form preferences with partners with whom they associate in various non-playful social contexts,^51^^,^^53^^,^^54^^,^^55^^,^^56^ in prior work we investigated whether juvenile male rats form partner preferences when playing in groups.^9^^,^^10^ In the first study, focal rats had a choice of a cage mate, a neighbor from a cage in which he could be seen, smelt, and heard, but not played with, and a stranger which he had never seen before. Even though the focal rats initiated play with all test subjects, they had a clear preference for the less familiar partners over cage mates.^28^ But what if all the potential partners in the test cage are strangers, will some be preferred over others? This led to the second experiment in which groups of six, completely unfamiliar partners were tested together. We found that, even among strangers, some rats form play partner preferences over the course of a 20 min play trial.^57^ Together, these findings suggest that, while less familiar rats are preferred, not all unfamiliar rats are equally valued as play partners. In a third line of experiments, we investigated whether rats have preferences within groups of familiar, co-habiting animals. As preliminary data suggested that they did, with rats leaving the vicinity of the closest rat to traverse the test cage to launch a playful attack on a distant, preferred partner,^9^^,^^10^ in the present study we further explored play partner preferences in groups of familiar rats.

**Figure 1:**
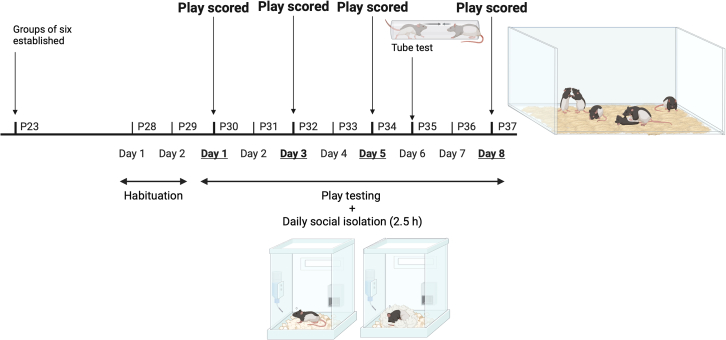
Experimental timeline The timeline of when the groups were established, habituated, and tested. Note that the rats were isolated and tested each day, but only days 1, 3, 5, and 8 were scored. The age of the rats, in postnatal days (P), is listed beside each hash in the timeline. Created with BioRender.com.

We investigated play in groups of six familiar juvenile male rats to verify whether co-habiting rats form play partner preferences. Moreover, as individual rats tend to be consistently high or low players with associated preferences in style of play,^10^^,^^27^^,^^30^ we predicted that play partner preferences should remain stable over the juvenile period. The reason for this prediction is that we hypothesized that rats select partners that most closely complement their individual play styles. Consequently, regarding mechanisms for partner selection, we predicted that the same individuals should remain as the most preferred partners and central to the play initiation network (as calculated by the animals’ eigenvector centrality score). Similarly, as turn taking and symmetry seems to be what influences the development of socio-cognitive skills in rats,^23^^,^^25^ we predicted that rats would prefer to play with partners that engaged in more turn taking and with whom they had more symmetrical play relationships, thus maximizing the occurrence of these experiences. Indeed, this might even lead to sub-communities forming within the group with complementary play partners playing more with one another. Therefore, play trials were run over eight consecutive days during the peak juvenile play period, sampling multiple days during this eight-day period (Figure 1). Partner preference predictions were tested using social network analysis methods.^10^^,^^46^^,^^47^

## Results

### Partner preferences

**Figure 2:**
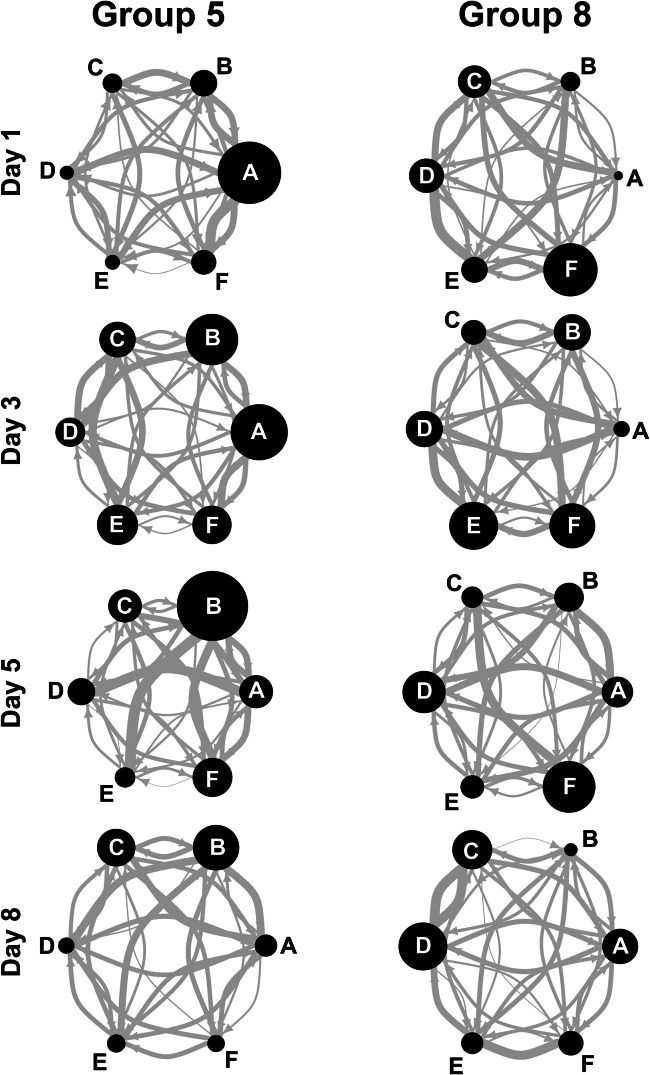
Partner preferences in two groups of rats Directed social networks are plotted illustrating the play preferences of two groups across the four test days. Each circle or node represents an individual in the group. The size of the node depicts the amount of play that individual initiated. The lines or edges connecting the nodes illustrates the proportion of play that individual directed toward the rats in the group. See also Figure S1.

Directed social networks, which plot the proportion of nape attacks initiated and received by all six members of the group, revealed that on any given day rats form partner preferences (Figure S1). These preferences are illustrated in the networks from two groups (Figure 2).

In addition to the visualized networks (Figures 2 and S1), we used Mantel tests to compare the play proportion matrices from each group on each day with a hypothetical equal distribution matrix. If the model found that matrices were correlated, that would indicate that the play relationships were random, and so animals did not have preferences. In other words, individuals within the group were the target of playful attacks as often as would expect by chance. We found that only two of the matrices (Group 3, Day 3 and Group 5, Day 8) were correlated with the hypothetical matrix (r = 0.55, *p* = 0.044; r = 0.49, *p* = 0.015, respectively).

**Figure 3:**
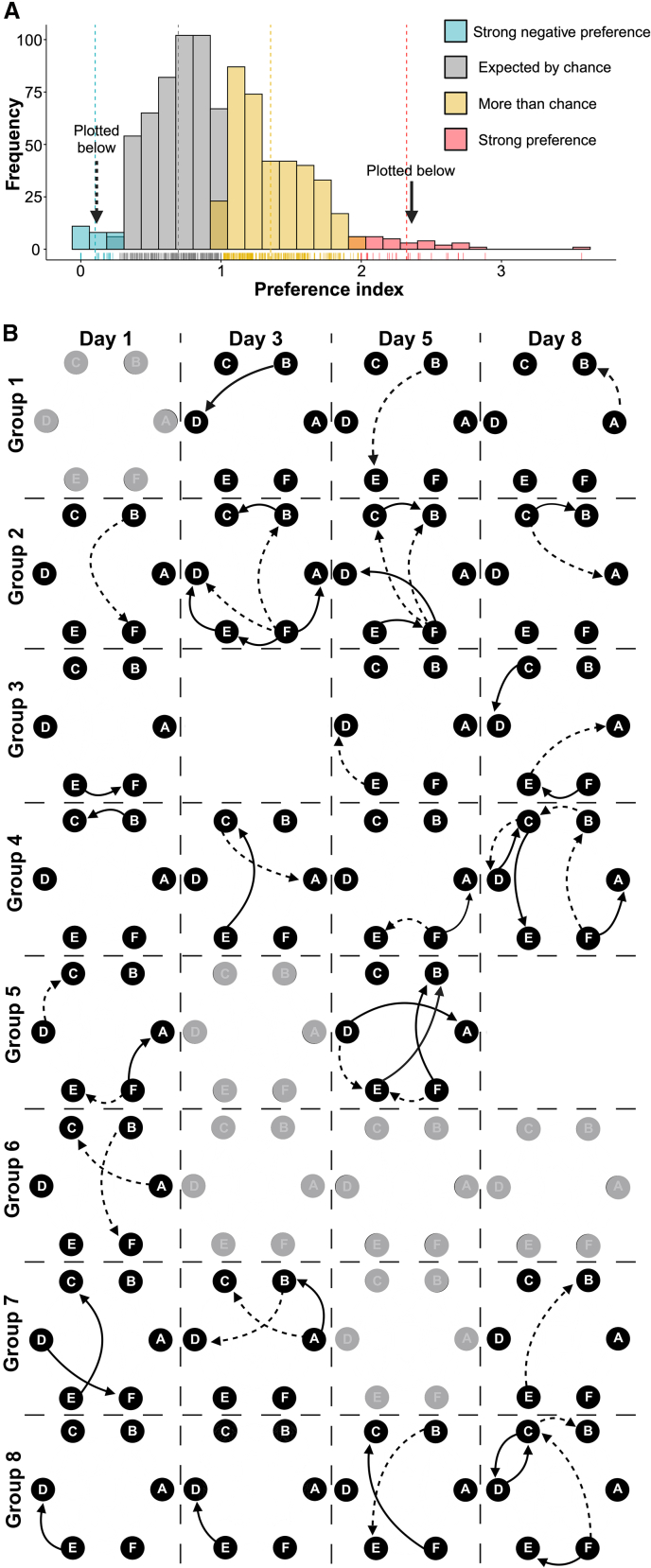
Not all rats are preferred, and not all rats have preferences The distribution of preference indices, or preference strength, for all possible combinations of initiator and recipient (A). Strong preferences are those that are ≥2, while weak preferences are ≤0.25 (A). Directed social networks are plotted illustrating the significant play preferences, of all eight groups across the four test days, except for Group 3, Day 3 and Group 5, Day 8 as we found the preferences on these days in these groups to be randomly distributed (B). Each node represents an individual in the group. The solid lines represent partners toward which playful attacks were launched significantly more than expected by chance and the dash lines the partners that received attacks significantly less than expected by chance.

Using Thompson’s play partner preference index calculation,^40^ we calculated the strength of partner preferences for each possible dyad, in each group, for each day, except for those excluded by the Mantel test (see above). With scores greater than 1 indicating play preferences that are greater than chance and those greater than 2 being strong preferences for a particular play partner,^40^ we found that there were 395 play relationships in which the preference scores were higher than expected by chance. Of the 395 play scores, 31 were equal to or greater than a score of two, suggesting some rats had strong preferences (Figure 3A). Conversely, we found that there were 27 relationships that had a score of 0.25 or less, suggesting that certain individuals within the group were not preferred as they were played with far less than expected by chance. These results are represented graphically in Figure 3B, showing only the strongly preferred and strongly not preferred relationships. These directed social networks illustrate that not all rats in the group express preferences and that who they prefer changes from day to day. Together, the results from the Mantel test and from the play partner preference calculations,^40^ suggest that the distribution of play is non-random, indicating that some rats are preferred or avoided when rats are playing in groups.

**Figure 4:**
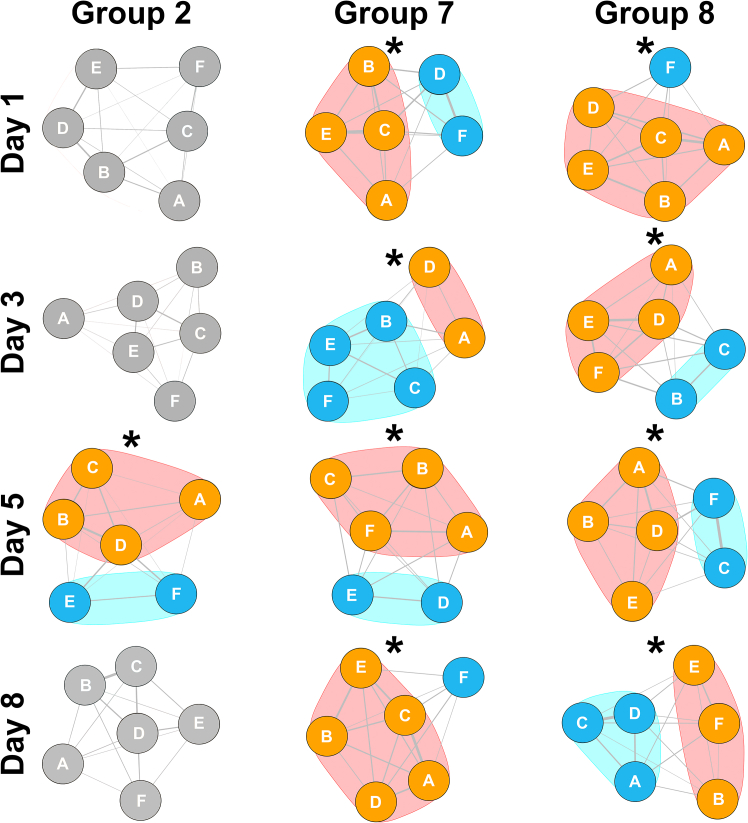
For three of the groups, play partner sub-groups form Network modularity or sub-group formation is plotted using undirected social networks. The lines or edges connecting the nodes represent the amount of play (and not the proportion of play) directed between pairs of rats. Asterisks represent days when the associations between sub-groups were significant. The sub-group associations are illustrated through the use of polygons.

Social network analysis revealed that only three of the eight groups formed sub-groups or communities (Figure 4). Of these three, sub-groups were only formed every day in two of the groups, and these sub-groups were not stable over the four days. Therefore, on most days, any partner preferences that were formed were assessed by factors specific to each day, not long-term group influences.

### Partner preference stability

**Figure 5:**
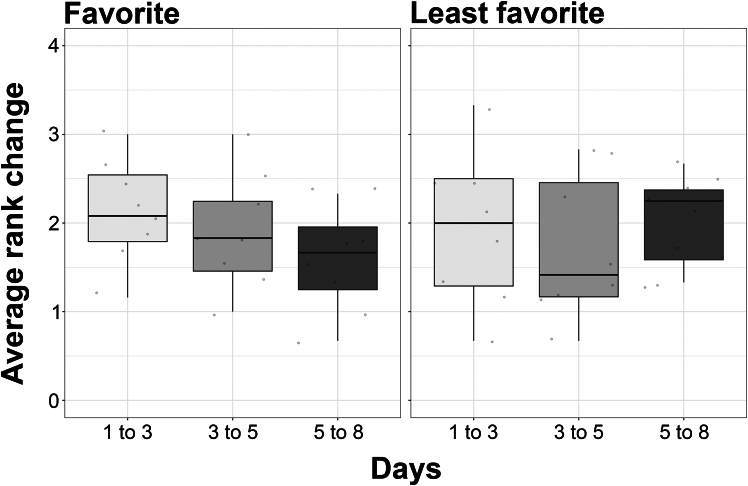
Partner preference stability The average change in rank, for each group, of the favorite (left) and least favorite (right) partner. The boxplots depict that between each of the test days, the preferences are unstable. A score of 0 would indicate no change in rank and partner stability, while a score of four would indicate a major change in rank and no partner stability. Data are represented as median +/− max and min.

Although rats formed preferences on any given day, these preferences, on average, were not maintained. The degree of partner change reveals that, on average, rats do not prefer to play with the same individual from day to day. Their favorite partner on a test day was typically their second favorite or middle favorite partner the previous day (Figure 5, left panel). Similarly, their least favorite was their second least favorite or middle favorite partner the previous day (Figure 5, right panel).

### Popularity

**Figure 6:**
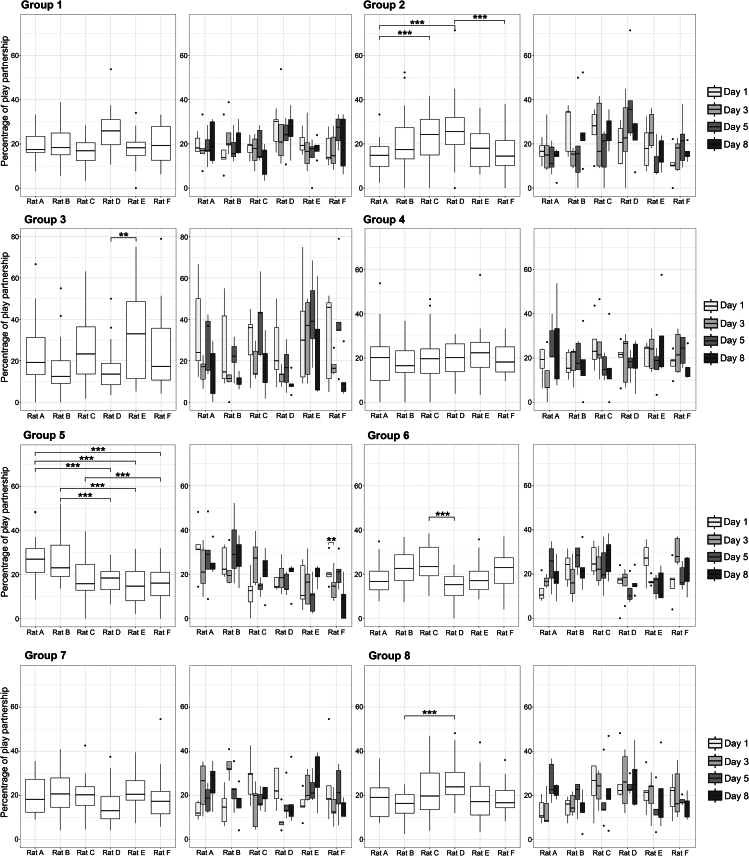
Popularity The overall preference by other group members for a given individual is plotted for each group. For some groups, certain individuals are consistently, and significantly, preferred over others. For each individual in every group, the preferences are plotted for each day. To test if the individuals that were significantly preferred or avoided changed from day to day, we used a repeated measures ANOVA. Data are represented as median +/− max and min with outliers plotted as individual points. ∗∗*p* < 0.01, ∗∗∗*p* < 0.001.

While some rats form partner preferences on any given day, repeated measures ANOVAs with a Bonferroni correction revealed that certain individuals in Group 2 (F(5,95) = 3.06, *p* = 0.013), Group 3 (F(5,95) = 3.31, *p* = 0.008), Group 5 (F(5,95) = 6.53, *p* < 0.0001), Group 6 (F(5,95) = 4.48, *p* = 0.0009), and Group 8 (F(5,95) = 2.96, *p* = 0.02) were consistently favored over others across the four test days (Figure 6).

Although certain individuals were preferred across the four days, it could be that their popularity on a given day, but not all days, drives this effect. To assess if certain individuals were equally popular each day, repeated measure ANOVAs with a Bonferroni correction were used to compare the individuals that were significantly preferred or avoided (Figure 6). The popularity of some rats fluctuated across testing days, whereas the popularity of other rats was stable. These models revealed that in Group 2, neither Rats A, C, D, nor Rat E changed from day to day. In Group 3, Rats D and E did not fluctuate in popularity from day to day. In Group 5, Rat F (F(1,3) = 5.47, *p* = 0.01), but not Rats A, B, C, E, changed from day to day. In Group 6, Rats C and D did not fluctuate from day to day, nor did Rats B and D in Group 8.

### Group and individual variation

**Figure 7:**
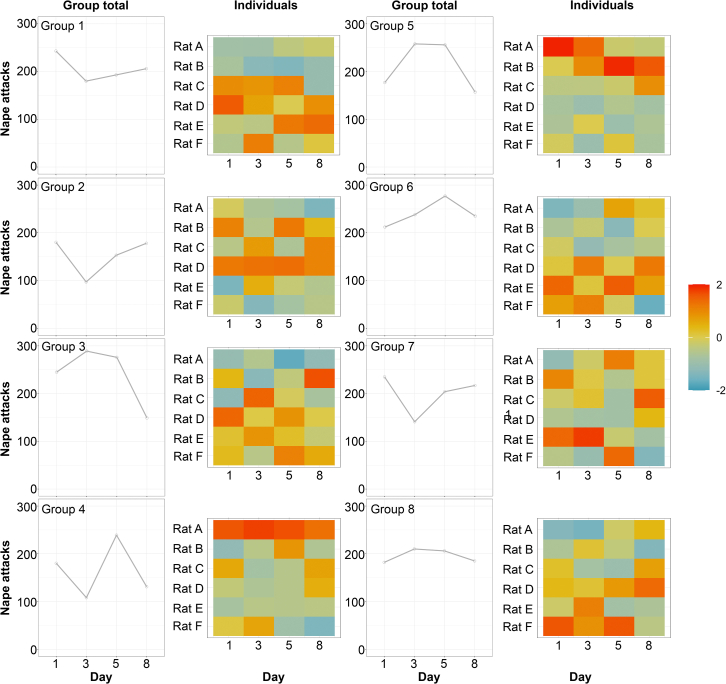
Group and individual variation The total number of nape attacks initiated by all group members is plotted for each of the four test days, for each group. Beside the total nape attack plots are heat maps illustrating whether individuals (*y* axis) on a given day (*x* axis), from their respective group, play above or below the mean of that day. Only deep red scores indicate individuals are playing significantly above the mean while only deep blue represents individuals playing significantly below the mean.

When summed for the four days, we found that groups did not show different frequencies of play interactions (F(7,21) = 1.67, *p* = 0.166). That is, there was no clear evidence for some groups being “high playing” and others “low playing”. A day-to-day analysis (Figure 7) revealed that the pattern of play varied among groups, with some groups playing at relatively consistent levels on all days (e.g., Group 8) and others fluctuating daily (e.g., Group 4). Corresponding heat maps of attacks initiated by individual members of groups (Figure 7) show that while some animals consistently play above the expected mean for the group (e.g., Rat A, Group 4), many animals fluctuate, playing above the mean on certain days and below on others (e.g., Rat C, Group 4).

### Potential mechanisms influencing preferences

#### Play frequency, style, and quality

Given the degree of variation in play across individuals (see Figure 2), a possible factor making some individuals more attractive play partners is that they initiate more nape attacks. We found that the number of nape attacks launched by a partner and directed toward the focal rat did not predict whether a partner was the preferred versus the not preferred partner (Table 1), as determined by the play partner preference scores (Figure 3A).^40^

**Table 1:** Comparisons of the non-preferred (*n =* 27) and preferred (*n =* 31) partners (dependent variable) with a binary generalized linear mixed model are presented below to compare various measures of the rats play behavior (predictor variables)

Fixed effect	Not Preferred	Preferred	Estimate	SE	z	p	OR	CI Lower	CI Upper
Intercept			−2.533	1.630	−1.555	0.120	0.079	−5.728	0.660
Partner play frequency	5.11 ± 0.63	7.84 ± 1.05	−0.252	0.145	−1.732	0.083	0.777	−0.537	0.033
**% no response**	**14.81 ±6.97**	**29.58 ±3.59**	**0.042**	**0.019**	**2.269**	**0.023**	**1.043**	**0.006**	**0.078**
% evasion	29.63 ± 8.96	31.61 ± 2.44	0.022	0.019	1.160	0.246	1.022	−0.015	0.058
% pin	11.11 ± 6.16	30.53 ± 3.72	0.030	0.020	1.557	0.119	1.031	−0.008	0.067
**% role reversal**	**2.38 ±1.43**	**47.28 ±7.15**	**0.136**	**0.052**	**2.598**	**0.009**	**1.146**	**0.033**	**0.239**

SE, standard error; OR, odds ratio; CI, confidence interval.

The frequency of play initiated by the partner and directed toward the focal rat, as well as the percentage (± standard error of the mean) of the various measures are presented along with the results of the model.

When we compared the percentage of defensive tactics used by preferred partners and non-preferred partners, we found that preferred partners were predicted to defend themselves more from a playful attack but did not use different defensive tactics (Table 1). Role reversals were predicted to occur more often with preferred partners (Table 1).

#### Dominance and weight

We found that dominance relationships and weight asymmetries did not predict whether a partner was the most versus least preferred favored partner (Table 2). The results of the tube test are tabulated in S2.

**Table 2:** Comparisons of non-preferred (*n =* 27) and preferred (*n =* 31) partners (dependent variable), with a binary generalized linear mixed model, are presented below to compare various measures of behavior, measured as a pair (symmetry, proximity, and dominance), as well as the difference in weight between pairs of rats (predictor variables)

Fixed effect	Not Preferred	Preferred	Estimate	SE	z	p	OR	CI Lower	CI Upper
Intercept			1.774	1.493	1.188	0.235	5.892	−1.152	4.700
**Play symmetry**	**0.51 ±0.06**	**0.75 ±0.05**	**−2.911**	**1.058**	**−2.750**	**0.006**	**0.054**	**−4.986**	**−0.837**
Proximity (sec)	292.65 ± 11.86	281.77 ± 10.46	−0.001	0.005	0.170	0.865	1.001	−0.009	0.011
Weight	−3.31 ± 3.57	−2.64 ± 3.35	−0.005	0.016	−0.296	0.767	0.995	−0.036	0.027
Dominance	1.92 ± 2.28	0.88 ± 1.51	−0.024	0.029	−0.820	0.412	0.976	−0.082	0.033

SE, standard error; OR, odds ratio; CI, confidence interval.

The percentage (± standard error of the mean) of the various measures are presented along with the results of the model.

#### Proximity

The amount of time that rats were in close proximity (within a body length) did not predict whether a partner was the most versus least preferred partner (Table 2). These data suggest that opportunity is not a key driver of partner choice, as on many occasions a rat left the company of group mates to traverse the cage to initiate play with another, more distantly located partner.

#### Partner availability

Given that when two rats are playing together, they are no longer available as potential partners, the partner preferences we detected could have been driven by partner availability. Therefore, we combed through the dataset and selected the nape attacks by the focal animals (i.e., those rats found to have a significant positive or negative preference based on the analyses presented above) when all five potential partners were available. On average, this resulted in 60.8% of the original attacks, occurring when all partners in the group were available. We used the previously found positive and negative play associations to predict whether the adjusted association should be above or below the median value. For preferred and non-preferred partners, the focal rats attacked as previously predicted (sign test: most preferred, *n* = 24/31, *p* = 0.0033; least preferred, *n* = 26/27, *p* < 0.0001). In other words, preferred rats were played with above the median, even when around 40% of the playful attacks were not included. Likewise, non-preferred rats were still avoided. Moreover, even when all five potential partners were not available, in over 98.8% of cases, the focal rats had at least two other rats available to direct their attacks. These analyses suggest that the partner preferences found were not simply byproducts of partner availability.

## Discussion

Given our preliminary findings,^9^^,^^10^ we predicted that rats living in groups should form play partner preferences, and that these preferences should remain stable over the juvenile period. Moreover, given that individual differences in playfulness tend to be stable over time and context,^27^^,^^30^^,^^31^ and that the quality of play, especially turn taking and symmetry, seems to be important in rewarding playful interactions,^58^^,^^59^ we predicted that rats should prefer high players as partners, who tend to play more reciprocally. Our findings support some, but not all, of these predictions. Many rats did exhibit play partner preferences (Figures 2 and 3). Preferred rats tended to engage in less defensive actions but engaged in more role reversals than unpreferred partners (Table 1). Contrary to our prediction, we found that partner preferences were unstable, with different partners being preferred on different days (Figure 3). In addition, there was considerable variation at a group level, with some groups showing clear preferences and others not, similar to the group variation in play preferences reported for domestic pigs (*Sus scrofa domesticus*).^38^

Together with our previous findings, the mechanisms used to form play partner preferences among juvenile male rats are becoming clearer. Weight and dominance asymmetries do not influence preferences when playing in groups in which all of the individuals are familiar (present study) or when all individuals are unfamiliar.^57^ However, when the choice is between a stranger and more familiar partners, these asymmetries do influence partner preferences, with a more dominant or larger stranger being a less attractive play partner.^28^ Still, this is a relatively minor influence as strangers that are closer in dominance to the subject are preferred over cage mates. Therefore, in the juvenile period, dominance asymmetries are, at best, modifiers under some partner choice situations, not a main determinant for partner choice. Unlike when rats are playing with unfamiliar partners,^28^^,^^57^ preferred partners among groups of familiar individuals are those which have a more symmetrical play relationship and engage in more role reversals.

A prediction that was not supported was that in groups of familiar animals the play preferences should be stable, as in wild Japanese macaques (*Macaca fuscata*), where play relationships are correlated over a long period of time.^37^ This was not what we found as preferences varied from day to day (i.e., they were unstable). Indeed, even at a group level, the sub-communities formed also varied from day to day, suggesting there is instability both in individual preferences and in group dynamics (Figure 4). However, the day to day changes in partner preferences were not random. If rats changed partner preference, they were most likely to switch to the second or third most preferred partners, not to the least preferred rats (Figure 5). Moreover, in many groups, while the status for an individual as the most preferred partner could change from one day to the next, it continued to rank among the preferred partners (Figure 6). That is, there tend to be clusters of high players within groups (Figure 2), with partner preferences switching among these group members over days. If this is the case, then two further issues need to be explored. First, what underlies the daily changes among the preferred partners, and, second, what is it about the least preferred rats that make them so undesirable?

### Potential factors associated with or influencing partner preferences

One possible factor influencing why the most preferred status can switch from one day to the next is that an individual alters its behavior. We found that the amount of play an individual engages in fluctuates from day to day (see Figure 7). So, the preferred high player on Day 1 may become less preferred on Day 3 as it initiates less play on that day, thus, leading the other high players in the group to pick a new “favorite partner.” Even though an individual high player (i.e., a rat that initiates relatively more nape attacks) may decline in its playfulness on any given day, overall, the same individuals tend to remain the group members initiating the most play in each group (Figure 7), suggesting that the same coterie of rats are likely the ones that remain among the preferred play partners. Furthermore, with changes in playfulness, subtle changes in how those rats play could tip the balance on the attractiveness of the previous favorite partner, so explaining the significant influence of defensive behavior and role reversals (Table 1, see also^28^^,^^57^). Alternatively, rats could be actively seeking diversity in play partners and interactions, making partner preferences relatively unstable over time (discussed further below).

Another potential explanation is that preferences are artificially driven by partner availability. That is, a rat may play with a given partner simply because all the other partners are busy playing. Our data, however, do not support this hypothesis. Rather, we found that in nearly all cases, an attacker had two or more potential partners to which to direct their play. Moreover, while scoring the attacks, casual observations indicated that, on many occasions, rats waited for their preferred partner to be available instead of attacking a non-preferred partner which was available.

A simpler, alternative explanation could be that, on any given day, rats that remain in closer proximity to one another are more likely to play together. This is unlikely because neither in this study, nor in two previous ones,^28^^,^^57^ in which we measured proximity, was there any evidence for an association between proximity and play partner preference. In addition, our observations during all these studies revealed that the least preferred rats would often be bypassed to reach a distant location and initiate play with one of the preferred partners.

It should be noted that although some group members were played with rarely, each animal in each group engaged in play with everyone in the group in almost every session (Figures 2 and S1). So, it is possible that play is being used, to some degree, as a means of maintaining social contact among group members, and that least preferred partners may still be selected for other kinds of social interactions. For example, allogrooming is used to manage social relationships among familiar animals in a home cage context.^60^^,^^61^ While allogrooming was rarely observed in the present study, given the pre-test session social isolation used to increase the likelihood of play, this is not surprising. Spontaneously occurring social interactions in groups of rats living in their home cages need to be monitored to determine whether trade-offs are being made between playing with group mates and grooming them. That is, a partner rarely played with may receive more allogrooming. So, while studies such as this one indicate that some individuals are not preferred as play partners, it cannot be assumed that they are completely ignored or treated as unattractive partners in other social contexts.

Regarding play fighting, rats may select partners in a way to regulate some preferred features of the play experience, such as some combination of the frequency of play^27^ and the balance between cooperation and competition during play.^5^ The role of homeostasis in shaping the amount and content of play is a relatively unexplored avenue of study.^27^^,^^62^ Moreover, the selection of play partners may also be influenced by a variety of subtle cues, such as odor, vocalizations, how they are contacted, or the complementarity of their actions relative to that of their partners.^3^^,^^63^^,^^64^^,^^65^ These mechanisms and factors remain to be explored.

It was clear that one or two rats per group never rose to being popular, with little play directed at them and they, in turn, directed little play to the others (Figures 2, 6 and S1). Whether these rats were “ostracized” (Table 1) because they were unattractive partners for some reason, or because they had a low motivation to play, remains to be determined. However, that each group had at least one outcast suggests that, in naturally occurring colonies of rats, not all rats may receive the same level of play experience, and this could affect the ability of the animals to gain the benefits typically associated with play.^9^ Indeed, naturally occurring variation in the amount of play experienced during the juvenile period has been found to have repercussions later in life in several species (e.g.,^66^^,^^67^^,^^68^^,^^69^ including rats^70^^,^^71^). The occurrence of natural variation in the play expressed by rats in the juvenile period,^27^^,^^30^^,^^31^^,^^52^ and the finding that the play experienced in the juvenile period, especially its quality, can affect the development of socio-cognitive skills and their underlying neural substrates,^22^^,^^23^^,^^25^^,^^72^^,^^73^^,^^74^^,^^75^ raises the possibility that rats may regulate partner choice as a way of gaining experiences that ensure appropriate developmental outcomes.^9^^,^^70^

### Maximizing two different kinds of experiences?

Specifically, it seems that it is the degree of symmetry and turn taking that individuals experience during play fighting as juveniles that influences the development of social skills and the dendritic arbors of the pyramidal neurons in the medial prefrontal cortex.^23^^,^^25^^,^^59^^,^^73^ These same features of the play experience also seem to be critical for keeping the playful behavior enjoyable,^76^ with rats engaging in behavior that facilitates turn taking.^77^^,^^78^ Given that young animals can create a social niche that is most beneficial to them,^79^^,^^80^^,^^81^ it is possible that niche creation may account for both our expected and unexpected findings about play partner preferences.

As expected, rats did prefer some partners over others, but unexpectedly, rather than direct all their playful attacks to one or two preferred partners, over the course of the test trial, they also attacked less preferred partners, including the least preferred ones (Figures 2 and S1). This is a pattern that is consistent with the study of play involving different group membership (e.g., all strangers or a mixture of strangers and familiar rats^28^^,^^57^). To gain the benefits derived from turn taking, juveniles appear to need to experience a minimum threshold level,^58^^,^^59^ with too little or too much leading to an excessive lack of symmetry in play fights, either of which can lead to detrimental developmental outcomes.^23^^,^^73^ Therefore, it is possible that an individual plays with suitable partners to reach that minimum threshold, so explaining the above expected levels of play directed at a coterie of group members (Figures 4 and 6). But what accounts for the play directed at the less optimal group members? Previous studies comparing being reared in a group versus being reared with a single partner during the juvenile period found that having more than one partner influences the dendritic arbor of the pyramidal neurons in the orbitofrontal cortex.^82^^,^^83^ Since damage to the orbitofrontal cortex in normally reared rats reduces their ability to modulate their responses with the identity of the partner in both playful and non-playful social interactions,^84^ it is possible that by interacting with multiple partners, rats indirectly train their orbitofrontal cortex to match responses to specific partners better.^9^ In contrast, the improvements in social skills and the anatomical changes in the medial prefrontal cortex can be achieved by playing with a single partner over the juvenile period.^20^^,^^21^^,^^22^^,^^23^^,^^25^^,^^74^^,^^82^^,^^85^^,^^86^ Damage to the medial prefrontal cortex in normally reared rats reduces their ability to adjust their movements to those of their partner in both playful and non-playful interactions, but does not disrupt the ability to modulate actions with the identity of the partner.^87^^,^^88^ These findings suggest that the play experiences train rats in how to modulate behavior with the actions of their partner.^9^ If so, then in a natural setting with multiple potential partners available, the rats should follow two simple rules: (1) play with partners that provide the minimum turn taking experiences needed to train the medial prefrontal cortex and (2) play with a sufficiently diverse number of partners to train the orbitofrontal cortex. The pattern of play partner preferences in the rats in the present study is consistent with this hypothesis and so worth further testing.

### Implications

With a push to study more ethologically relevant behaviors and test animals under more naturalistic conditions,^89^^,^^90^^,^^91^ we propose that, depending on the question being asked, group play may be a more suitable paradigm to explore juvenile social play and social development.^32^

With that said, it is clear from our results that the choice of partners should be provided to rats, and that rats should be tested on more than one day as individual idiosyncrasies in play change day to day. For example, Figure 2 reveals that while certain animals maintain a relatively stable play frequency (e.g., Rat D), others vary greatly from session to session (e.g., Rat A). Therefore, scoring only one day may provide a misleading snapshot of Rat A’s behavior. While this requires more effort, we do not feel this is a limitation but is instead beneficial, especially when phenotyping individual differences and studying social development. Indeed, these individual differences more closely resemble those of human children engaging in play.^92^

Elucidating individual differences with the group play paradigm may prove particularly useful when studying rat models of diseases and psychological disorders.^93^^,^^94^ By giving the rats a choice of partners, certain individuals may be avoided, revealing nuanced social deficits. For example, if groups of three are tested, in which two of the three are “control” rats and one is an “experimental” animal (e.g., a disease or social deficit model strain), determining the frequency and style in which a control plays with the control partner versus the experimental partner may reveal the severity of the deficits.^32^^,^^95^ However, if a control rat is playing under dyadic conditions, the rat has no choice but to play with its prescribed partner, and this could potentially mask such deficits. This is particularly true when the rats have been socially isolated for a period of time, a standard practice of studying play,^10^^,^^96^ and so are highly motivated to play.

### Limitations of the study

One limitation of this study is that it was limited to males. We used male rats in this experiment as dominance hierarchies are more prevalent in males^50^ and these hierarchies may have influenced their partner selection. However, dominance asymmetries did not influence the selection of play partners, and so females would likely express partner preference patterns similar to males.^28^ Given that females are sensitive to the identity of their play partner,^97^ future studies should use females as well, as these may highlight some of the factors not readily discerned in all male groups. Additionally, studying mixed-sex groups or groups of mixed kin and non-kin animals may be worthwhile as this would more accurately reflect the natural variation in play partners to which wild rats would have access. Indeed, in group living, Belding’s ground squirrel (*Urocitellus beldingi*) juveniles prefer to play with relatives rather than neighbors, but have no preference for full versus half siblings.^41^ Even so, specific individuals are preferred over others.^42^ Whether kin based preferences are formed in wild and/or domesticated rats remains to be determined.

Another potential limitation is that the rats came from general holding pens from Charles River, so some of the rats may have been related. While we have no way of knowing how many, if any, were related, this could potentially drive some of the play partner preferences present. Future studies should examine whether rats have preferences for kin, using in-house breeding to ensure that the relatedness of the group members are known, and compare those results to other communally living rodents, such as Belding’s ground squirrels.

While we chose to sample play in the typical experimental fashion (i.e., animals are isolated and then tested in an enclosure), to be sure that the preferences observed in these snapshots reflect the rats’ preferences throughout the day, home cage records are necessary. Indeed, we are currently analyzing the data from such a project. However, since most studies of play in rats involve play sessions that occur once a day and only last between 5 and 10 min, we wanted to use a similar approach here. That way we could determine if, during that time frame, partner preferences emerge. Whether they are maintained over a long period of time and throughout the day remains to be determined.

Finally, the tube test, though frequently used, may not provide accurate results as to the animals’ dominance relationships. Instead, “wins” and “losses” during the tube test could simply arise due to differences in motivation, behavioral strategies, or size. As this was our only measure of dominance in the present study, we cannot be sure that we effectively tested the influence of dominant-subordinate relationships within the groups of rats. These findings need to be corroborated using other measures of dominance.

### Conclusions

The results from this study demonstrate that rats have partner preferences when playing with familiar cage mates. However, these preferences vary from day to day. While dominance and weight asymmetries do not influence preferences, the likelihood that the partner defends itself, the degree of symmetry between rats and the degree to which they take turns was correlated with partner preference formation. In most of the groups, certain individuals were significantly preferred over others each day, suggesting that some rats are more *popular* than others. Together, these data suggest that animals are not at the mercy of their environment but can create a social niche that is preferred. The created social niches may be selected to maximize both the high quality and diversity of play experiences. By giving the rats a choice of partners and so a choice in experiences and quality of play, we let the rats *tell us* which aspects of their play they value (i.e., play frequency, style, quality, or the individual). In turn, this more naturalistic approach to studying play and social behaviors offers insights into group dynamics and nuanced individual differences which may, in turn, offer new insights when studying rat models of disease and development.

## Resource availability

### Lead contact

Further information and request for resources should be directed to the lead contact, Jackson R. Ham (jackson.r.ham@gmail.com).

### Materials availability

This study did not generate new unique reagents.

### Data and code availability

• All the raw data used for statistical analyses have been deposited on figshare and are publicly available as of the date of publication (https://doi.org/10.6084/m9.figshare.28781525).
• The code used for statistical analyses has been deposited on figshare and are publicly available as of the date of publication (https://doi.org/10.6084/m9.figshare.28781525).
• Any additional information required to reanalyze the data reported in this paper is available from the lead contact upon request.

## Acknowledgments

We thank Vivien C. Pellis, David R. Euston, E.J. Marijke Achterberg, and three anonymous reviewers for their comments and valuable advice on earlier drafts. The research was supported by grants from the Natural Sciences and Engineering Council of Canada (NSERC) (S.M.P. [grant number 2018-03706 and 2024-03978] and J.R.H. [CGS D]).

## Author contributions

J.R.H.: Conceptualization, formal analysis, methodology, visualization, writing – original draft, writing – review and editing. S.M.P.: Conceptualization, formal analysis, methodology, supervision, visualization, writing – original draft, writing – review and editing, funding acquisition.

## Declaration of interests

The authors declare no competing interests.

## STAR★Methods

### Key resources table

**Table:** 

REAGENT or RESOURCE	SOURCE	IDENTIFIER
**Deposited data**		

Analyzed data	This paper	Figshare (https://doi.org/10.6084/m9.figshare.28781525)

**Experimental models: Organisms/strains**		

Rat/Long Evans (RRID:RGD_2308852)	Charles River Laboratories	N/A

**Software and algorithms**		

R Studio	R Studio	https://cran.r-project.org

### Experimental model and subject details

#### Animals

We purchased 48 Long Evans male weanlings from Charles River Laboratories (Kingston, NY, USA) which arrived at the Canadian Center for Behavioral Neuroscience at 22 days of age. On arrival, we housed the rats in Tecniplast GR1800 double decker cages in groups of 6, resulting in a total of 8 groups. The floor was covered in corncob bedding and food and water were available *ad libitum*. The rats were housed on a 12-h light-dark cycle (lights on at 7:30 a.m.) and maintained at a constant temperature of 21°C–23°C and humidity. All care and testing procedures were reviewed and approved by the University of Lethbridge Animal Welfare Committee (protocol #1809) in compliance with guidelines from the Canadian Council for Animal Care.

While studying play in groups of rats is still in its infancy, we chose to form groups of six as this would allow for a great deal of selection and variation, while still being manageable to score. By constructing groups of six, the rats could choose to play with five different individuals, and even if a pair was already playing, rendering them unavailable, an individual still had the choice between three other animals.

### Method details

#### Apparatus

We tested the juvenile male rats in a large Plexiglass enclosure (80 cm × 80 cm x 50 cm). Corncob bedding was used to cover the floor of the enclosure sufficiently. We used an ExmourRS 4K Sony Handycam for video recording the play sessions. We placed the camera at a 90° angle above the enclosure.

#### Procedure

Before play testing began, starting at 28 days of age, we habituated the rats to the test enclosure for 10 min per day, with their cage mates, in red light, for two, consecutive days (Figure 1). At 30 days of age, testing began. Prior to the play sessions, we socially isolated the rats (food and water was available *ad libitum*) for 2.5 h to increase their playfulness,^27^ and the groups of six cage mates were placed in the enclosure, in red light, for 20 min and filmed. We repeated this testing procedure over eight consecutive days, over the peak play period.^10^^,^^98^ Indeed, this is the period when the play influences both behavior and brain development.^9^ Between each trial, we cleared the cage with Virkon and we replaced the bedding. To identify the individuals within the group, we drew tail patterns on each rat with a permanent marker pen (Sharpie).

#### Behavioral analysis

Days 1, 3, 5, and 8 were analyzed and compared. The trials on days 2, 4, 6 and 7 were run in case of mishap in the targeted trials (such as camera malfunction). As all targeted trials were successful, only those were scored and compared across all eight groups. Alternating days were selected so we could sample throughout the peak juvenile play fighting period, while still determining whether rats form preferences and, if they did, whether those preferences remain stable over the peak play period. We analyzed the 20 min video recordings using both normal speed and frame-by-frame analysis to score the microstructure of the rat play.^10^^,^^96^ We scored each video six times, following one focal rat at a time, noting the attacks initiated and directed toward each of the individuals within the group. A playful attack was scored when the snout of a rat made contact with the nape of another rat, as this is the target in rat rough-and-tumble play in around 90% of playful attacks.^96^ In addition, if the playful attack was launched but was evaded before contact could be made, this too was scored as a playful attack.

A rat defending itself in play can either defend its nape or ignore the attack and continue with its ongoing behavior (e.g., grooming, exploring, playing with another partner). In this case, the response to an attack was scored as ‘no response.’ If the attacked rat defended itself, it could do so by either evading its attacker (e.g., swerve, jump or run away) or by engaging in a facing defense in which the defending rat pivots to face its attacker. Once facing its attacker, the playful defense could result in wrestling on the ground if the defender rolled onto its back and was pinned there by the attacker, or in an upright ‘boxing’ position if the defender remained standing on its hind feet. In addition, we measured the number of role reversals (i.e., when the defender launches a successful counterattack and becomes the attacker).^10^ Measuring the number of role reversals provides a gauge on the quality of play as turn taking is a fundamental aspect of keeping play cooperative and reciprocal.^6^^,^^78^

Another marker of reciprocity, and so the quality of play, is the degree of symmetry in the playful actions of the partners.^73^^,^^99^^,^^100^^,^^101^^,^^102^ To assess the degree of symmetry in playful attacks, we subtracted the number of attacks by one partner from those by the other, and the absolute difference was divided by the sum of the nape attacks by both partners (see equation below). This value is subtracted from 1, with values closer to 1 indicating a high degree of symmetry, while values closer to 0 indicate a high degree of asymmetry.^10^

$$\text{Symmetry}=1-\left(\frac{\left|\left(\text{Rat}\;A\;\text{playful}\;\text{attacks}\to \text{Rat}\;B\right)-\left(\text{Rat}\;B\;\text{playful}\;\text{attacks}\to \text{Rat}\;A\right)\right|}{\sum \text{Rat}\;A\;⇌\;\text{Rat}\;B\;\text{play}}\right)$$

To assess the relative dominance among animals, we employed the tube test.^103^^,^^104^ Although the animals were juveniles, at this age, male rats start forming dominance relationships which can affect how they play with one another.^49^^,^^105^ We ran the tube test at 35 days of age using a Plexiglas tube that was just large enough to allow a rat through, but not so large that the rat could turn around or a second rat could squeeze by (19.5 cm in length and 4.5 cm in diameter). The tube test compared each possible pairing in the group of six. Between testing each pair, the tube was cleaned with Virkon. For each pair, a ‘winner’ and a ‘loser’ was designated based on who out of the pair stayed in the tube. In other words, the winner pushes the loser completely out of the tube (i.e., all four paws of the ‘loser’ outside of the tube). The winning rat was given a score of one. If both rats remained in the tube for 60 sec, this was considered a tie, and no point was awarded. Every pairing was tested consecutively five times. We used the sum of wins from each trial to rank the rats from most dominant to least, with the most dominant rat having the highest score.

Given that rats typically play in pairs, even if multiple individuals are present, a factor influencing partner preferences could be partner availability. That is, rats might not have the choice of playing with a preferred partner, but instead whichever rat is available. To assess if partner availability influenced partner selection, we calculated how many nape attacks occurred when two or more partners were available. Additionally, we calculated how many nape attacks occurred when all five partners were available.

One possible explanation for partner preferences is social proximity, in which rats play with whoever is closest and so most convenient to play with. Alternatively, rats may seek out a particular partner with whom to play within the box. As such, we measured the time the rats spent in close social proximity. Time spent in social proximity was measured if they were within one body length of each other.^28^^,^^57^ As some of the time spent in close proximity was due to the animals playing with one another, we subtracted the time the individuals spent playing with each other from the total time they were in proximity to each other.

### Quantification and statistical analysis

#### Statistical analysis

To visualize partner preferences, we constructed social networks using the *igraph* package^106^ in R Studio.^107^ We plotted directed social networks, with the size of the nodes or vectors representing the total amount of play (frequency) each rat initiated. The thickness of the edges or lines connecting the nodes represents the proportion of nape attacks each rat directs toward its partner. The proportion of play, and not the frequency, was plotted so that comparisons of preference strength could be made despite there being a considerable degree of variation in the amount of play each rat initiated.

We evaluated the social networks of each group, for each day, for randomness using a Mantel test^108^ in R with the *ade4* package.^109^ To do so, we constructed ‘real’ data matrices using the proportion of play directed toward each of the five potential partners on each day. We used the proportion of play to account for variation in play frequency among individuals. We then created a ‘hypothetical’ matrix consisting of the expected values if the rats played with each partner equally. If the distribution of play among partners was random, the ‘real’ and ‘hypothetical’ matrices should be correlated. Both matrices were transformed into distance matrices. We performed 9999 permutations to generate a null distribution and assess the observed correlation between the real and hypothetical datasets. If, on a given day, a group’s proportional play matrix was correlated with the hypothetical data, those days were excluded from further partner preference analyses (i.e., preference index scores, generalized linear mixed model comparisons).

When the two matrices differed significantly (Mantel test), we calculated a preference index score for each possible dyad using Thompson’s calculation^40^ (see below). The index scores range from 0 to infinity and indicate the strength of preference for each of the potential play partners within the group.^40^

Iij=BijBik−1

Values greater than 1 indicate more play interactions than expected by chance, while those equal to or above 2 indicate a strong preference.^40^ Scores equal to or below 0.25 indicate fewer interactions than expected by chance, and so likely avoidance of those individuals. The formula, where *k* is the number of rats in the group (6), *B*_*ij*_ is the number of nape attacks initiated by the *i*^*th*^ rat with *j*^*th*^ rat as the recipient.^40^ *B*_*i*_ is the total number of nape attacks initiated by the *i*^*th*^ rat.^40^ After calculating the preference index scores, their distribution was plotted using the package *ggpubr*.^110^

To illustrate the strong partner preferences on any given day, as determined by play partner index scores (described above), directed social networks were created. Only strong positive and negative preferences are depicted in these networks (play index scores ≤0.25 or ≥2), with solid edges depicting a preferred partner and hashed edges indicating an unpreferred partner.

We constructed additional networks to assess if sub-groups or -communities were formed.^111^ When sub-groups are present, networks have non-zero modularity scores varying between −1 and +1. When sub-groups or communities are not detected, or, in other words, when the distribution of edges are not different from what would be expected in a randomized network, the modularity values are equal to zero. When scores are equal to zero, this suggests that no sub-groups were formed. Where plotted, asterisks represent days when the associations between sub-groups were significant.

To calculate the stability of partner preferences, we calculated the degree of change in favorite and least favorite partner for each rat between days 1–3, 3–5, and 5–8. To calculate a change in rank, the rank on each day was subtracted from the prior day. For example, if Rat A’s favorite partner on Day 3 (rank = 1) was his least favorite partner on Day 1 (rank = 5), this would result in a large change in rank (rank 5 – rank 1 = 4), this being the maximum change in rank. However, if the rat changes from playing with his favorite rat to his second favorite rat (rank 2 – rank 1 = 1) between Days 1 and 3, this would be a small rank change. If the rat’s favorite partner remained the same, the rank change score would be 0 as there was no change. With this simple calculation, we assessed whether the preferences were stable between days and if not, to what degree they had changed. We plotted the distribution of changed values in R using *ggplot2.*^110^

We used repeated measures ANOVAs with Bonferroni corrections to compare if certain rats within a given group were played with more often than others (i.e., if some rats are more popular than others). The overall preference by other group members for a given individual was tested by comparing the percentage of play directed to each rat in the group. Although certain individuals might be preferred across the four days, it could be that their popularity on any given day, but not all days, drives this effect. As such, if we found that certain individuals were significantly preferred across the four test days, a repeated measures ANOVA with a Bonferroni correction was used to test if the percentage of play directed toward a preferred rat changed from day-to-day. In both cases, where plotted and when significant, asterisks are used to indicate significance (∗∗*p* < 0.01, ∗∗∗*p* < 0.001).

As play varies from individual to individual,^28^^,^^30^ we predicted that some groups might also vary, with some being high playing while others were not. To visualize group variation, we plotted the total play observed in each group (i.e., the sum of all playful attacks by each of the six rats). We used a repeated measures ANOVA with a Bonferroni correction to compare whether certain groups consistently played more than others across the four days. To assess whether certain individuals within the group played more than others, we calculated *z*-scores for the total play initiated on any given day. In doing so, we assessed whether certain individuals consistently played above or below the mean. To visualize the *z*-scores, heatmaps were plotted.

Other factors, such as play quality and style, were modeled with a generalized linear mixed model (GLMM) using the “glmer” function from the *lme4* R package.^112^ More specifically, we tested whether variation in the frequency of playful attacks launched by the partner rat and directed toward the focal rat, play style (i.e., the percentage of responses to playful attacks, the percentage of attacks defended by evasion or rolling into a supine pin position), and play quality (i.e., percentage of role reversals) predicted partner preference. To model these aspects of play style and quality (predictor variables), the partner preference, using only the not preferred (*n =* 27) and preferred partners (*n =* 31) (as determined by the play partner preference index,^40^ see Figure 3B for the inter-animal relationships tested), was set as the dependent variable. Given that play partners could either be “not preferred” or “preferred,” a binary GLMM was used. We set the initiating rat’s respective identification number as a random error term to account for the repeated measures across days. Thus, we compared the frequency of play initiated by the partner and percentage of response, evasion, pin, and role reversals (predictor variables) between not preferred and preferred partners (dependent variable). A total of 58 lines of data were compared.

We also tested pair measures using a GLMM. In our second model, we tested whether play symmetry, relative differences in weight and dominance, and social proximity were associated with our dependent variable (partner preference). As with the first model, we modeled only the play between not preferred (*n =* 27) and preferred partners (*n =* 31), which was set as the fixed effect, with the initiating and receiving rat’s respective identification number set as a random error term to account for the repeated measures. A total of 58 lines of data were compared. For both models, Wald’s confidence intervals are reported.

To correct for partner availability, the number of nape attacks launched when all partners were available was subtracted from the total number of nape attacks originally scored for significantly preferred and not preferred partners. If these partners were truly preferred or avoided, they should still be above or below the median despite not correcting the other, non-significant play relationships. We used sign tests to assess if preferred and non-preferred rats were attacked above or below the median, respectively, after correcting the number of nape attacks for availability.

All the figures generated, and statistical analyses reported, were done using R Studio.
