# Supplementary material for: Groups of familiar male rats form unstable partner preferences when play fighting during the juvenile period
Source: iScience. 2025 May 2;28(6):112562. doi: 10.1016/j.isci.2025.112562 (PMC12144427; doi:10.1016/j.isci.2025.112562)
Supplement: Document S1. Figure S1 and Table S2 [file mmc1.pdf]

iScience, Volume *28*

Supplemental information

Groups of familiar male rats form unstable partner preferences when play fighting during the juvenile period

Jackson R. Ham and Sergio M. Pellis

Supplemental Material

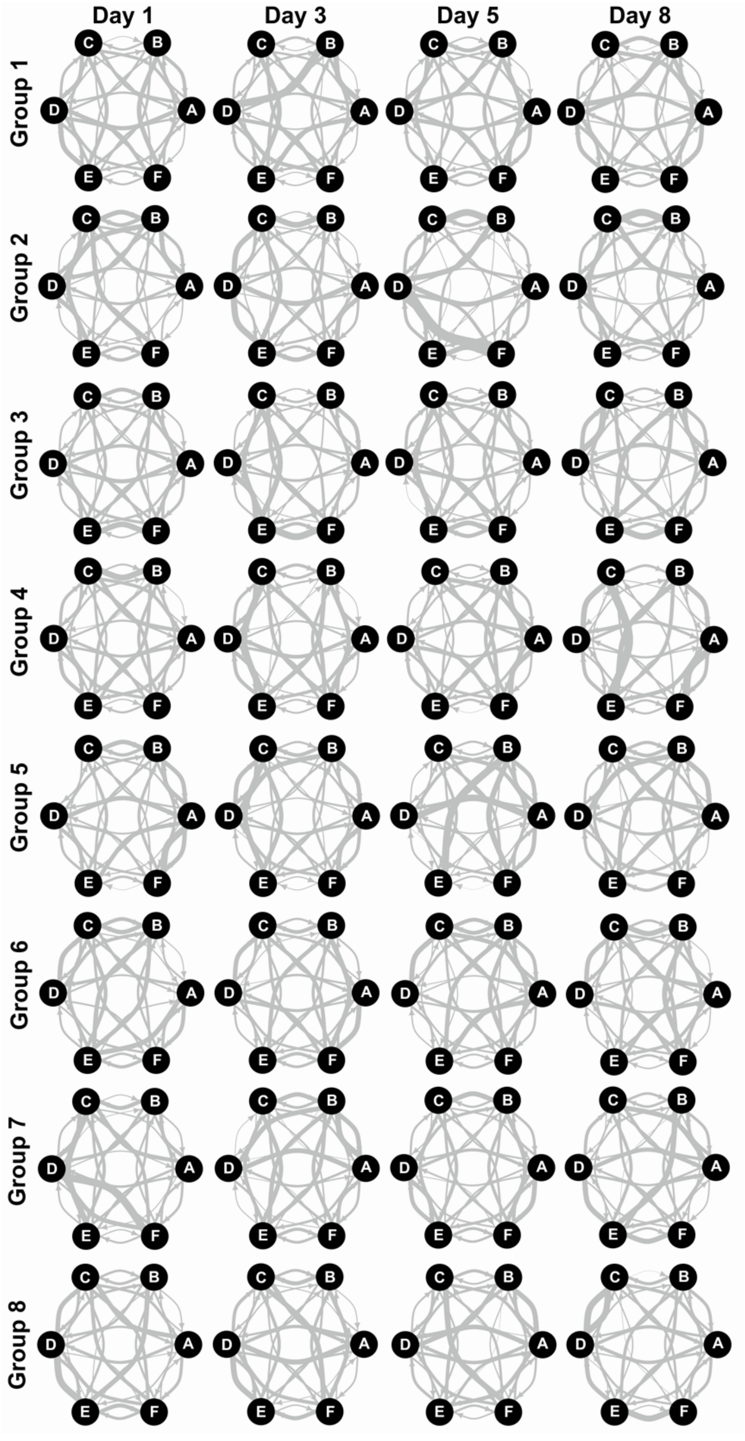

Figure S1. Directed social networks for each group on all four days. The circles or nodes represents the individual rats in the groups. The lines or edges connecting the nodes illustrates the proportion of play that individual directed towards the rats in the group.

Table S2. Results from the tube test, for each group. For every possible pair in the group, five trials were conducted. The maximum number of trials a rat could have been 25 and would indicate dominance over all individuals while a 0 would indicate complete subordinance. Rows represent wins while columns represent losses.

Group one

Rat	A	B	C	D	E	F	Wins
A	X	0	4	3	1	1	9
B	3	X	2	3	4	1	13
C	1	2	X	3	2	0	8
D	2	1	2	X	5	0	10
E	3	0	2	0	X	1	6
F	3	4	4	5	4	X	20

Group two

Rat	A	B	C	D	E	F	Wins
A	X	3	2	0	0	0	5
B	2	X	0	0	0	0	2
C	3	5	X	1	1	0	10
D	5	5	4	X	5	0	19
E	5	5	4	0	X	5	19
F	4	5	5	5	0	X	19

Group three

Rat	A	B	C	D	E	F	Wins
A	X	3	4	5	3	2	17
B	2	X	2	5	3	5	17
C	1	3	X	2	1	1	8
D	0	0	2	X	4	2	8
E	2	2	4	0	X	2	10
F	3	0	3	3	2		11

Group four

Rat	A	B	C	D	E	F	Wins
A	X	1	2	0	0	0	3
B	4	X	5	1	2	5	17
C	2	0	X	0	1	3	6
D	5	4	5	X	1	3	18
E	5	3	4	4	X	5	21
F	5	0	2	2	0	X	9

Group five

Rat	A	B	C	D	E	F	Wins
A	X	5	5	2	2	4	18
B	0	X	4	2	2	3	11
C	0	1	X	0	1	3	5
D	3	3	5	X	4	5	20
E	3	3	4	1	X	3	14
F	1	2	1	0	2	X	6

Group six

Rat	A	B	C	D	E	F	Wins
A	X	0	2	5	4	5	16
B	5	X	4	5	5	5	24
C	3	1	X	5	3	4	16
D	0	0	0	X	0	1	1
E	1	0	2	5	X	2	10
F	0	0	1	3	3	X	7

Group seven

Rat	A	B	C	D	E	F	Wins
A	X	1	1	2	4	0	8
B	4	X	5	5	5	3	22
C	4	0	X	2	5	2	13
D	3	0	3	X	5	2	13
E	1	0	0	0	X	1	2
F	5	2	3	3	4	X	17

Group eight

Rat	A	B	C	D	E	F	Wins
A	X	4	0	5	4	4	17
B	1	X	0	1	2	3	7
C	5	5	X	4	3	4	21
D	0	4	1	X	3	4	12
E	1	3	2	2	X	2	10
F	1	2	1	1	3	X	8
